# The dynamics of the nursing and midwifery professions: Initial findings from analysis of longitudinal registrant data

**DOI:** 10.23889/ijpds.v8i2.2310

**Published:** 2023-09-14

**Authors:** Iain Atherton, Michelle Jamieson

**Affiliations:** 1 Edinburgh Napier University, Edinburgh, United Kingdom; 2 Scottish Centre for Administrative Data Research, Edinburgh, United Kingdom

## Objectives

To ascertain geographical differences in retention of nurses and midwives across the United Kingdom using registrant data.

## Methods

The Nursing and Midwifery Council (NMC) are responsible for holding a register of all nurses and midwives in the United Kingdom. Registrants are required to revalidate every three years. Linking together resulting data creates a longitudinal dataset that follows registrants over time. The NMC is providing anonymised data through the ONS Safe Researcher Service (SRS). Data sharing agreements have been signed off and data is in process of being ingested by ONS. Initial analysis will focus on geographical differences in retention by for nurses by field of practice (adult, mental health, children, and learning disability) and midwifery.

## Results

There are estimated to be around 750 thousand nurses and midwives currently registered. Processes used to take this work forward will be described including public and stakeholder engagement. Early findings will be presented comparing demographic profiles of the professions and, for nursing, fields of practice in 2018 and 2021. Cox proportional hazard models will enable comparison of geographical differences in retention between England, Scotland, Wales, and Northern Ireland.

## Conclusion

Registrant data provides a basis that can inform policy. This is especially important given current challenges with regard to recruitment and retention in the nursing and midwifery professions. Future work will be outlined that will utilise registrant data including linkage to other administrative and census data sources.

